# Potent Blockchain-Enabled Socket RPC Internet of Healthcare Things (IoHT) Framework for Medical Enterprises

**DOI:** 10.3390/s22124346

**Published:** 2022-06-08

**Authors:** Abdullah Lakhan, Tor Morten Groenli, Arnab Majumdar, Pattaraporn Khuwuthyakorn, Fida Hussain Khoso, Orawit Thinnukool

**Affiliations:** 1 Department of Computer Science, Dawood University of Engineering and Technology, Karachi 74800, Pakistan; abdullah.lakhan@duet.edu.pk (A.L.); fidahussain.khoso@duet.edu.pk (F.H.K.); 2 Mobile Technology Lab (MOTEL), Department of Technology, Kristiania University College, Kirkegata 24-26, 0153 Oslo, Norway; tor-morten.gronli@kristiania.no; 3 Faculty of Engineering, Imperial College London, London SW7 2AZ, UK; a.majumdar@imperial.ac.uk; 4 College of Arts and Technology, Chiang Mai University, Chiang Mai 50200, Thailand; pattaraporn.khuwuth@cmu.ac.th

**Keywords:** socket, RPC, SOA, CORBA, RMI, IoHT, blockchain, client–server

## Abstract

Present-day intelligent healthcare applications offer digital healthcare services to users in a distributed manner. The Internet of Healthcare Things (IoHT) is the mechanism of the Internet of Things (IoT) found in different healthcare applications, with devices that are attached to external fog cloud networks. Using different mobile applications connecting to cloud computing, the applications of the IoHT are remote healthcare monitoring systems, high blood pressure monitoring, online medical counseling, and others. These applications are designed based on a client–server architecture based on various standards such as the common object request broker (CORBA), a service-oriented architecture (SOA), remote method invocation (RMI), and others. However, these applications do not directly support the many healthcare nodes and blockchain technology in the current standard. Thus, this study devises a potent blockchain-enabled socket RPC IoHT framework for medical enterprises (e.g., healthcare applications). The goal is to minimize service costs, blockchain security costs, and data storage costs in distributed mobile cloud networks. Simulation results show that the proposed blockchain-enabled socket RPC minimized the service cost by 40%, the blockchain cost by 49%, and the storage cost by 23% for healthcare applications.

## 1. Introduction

Socket programming is an essential application programming interface (API) that designs the different client–server healthcare applications in practice [1]. The healthcare application was developed based on many existing architectures [2], such as the service-oriented architecture (SOA), the common object request broker architecture (CORBA), the remote procedure call (RPC), and remote method invocation (RMI) [3]. Java is a giant language that designs different healthcare applications based on existing architectures [4]. The Java virtual machine (JVM) is a cross-platform interpreter that can run socket classes on various platforms to support users’ healthcare applications, such as real-time heartbeat monitoring, blood pressure monitoring, ECG live streaming, EEG framing, and others that are designed based on the mentioned architectures [5,6,7,8].

Data security between these architectures, such as CORBA, SOA, RPC, and RMI, has not been implemented to support advanced enterprise medical applications [9]. There are many computing nodes and data transmission nodes involved in these architectures. Therefore, node-to-node validation of data has been missed in these architectures [10]. Blockchain is an emerging technology that offers node-to-node validation, immutable transaction transmission, and self-healing security processes for applications [11]. Refs. [1,2,3,4] suggested using the SOA architecture based on the AES-enabled security mechanism for medical applications. However, these centralized security mechanisms have interoperability issues on different platforms. Refs. [5,6,7,8,9] suggested using the RMI-based security mechanism for the medical care applications in the fog cloud network. However, physical layer security suffered in these models when cyber-attacks were attempted on the different devices in the architecture. The RPC enabled the two-way security schemes that were suggested in [10,11,12,13]. The objective was to minimize the security risk at the network and physical layers in the architecture. To the best of our knowledge, the blockchain-enabled socket programming RPC architecture that supports node-to-node security and optimizes medical enterprise applications’ cost processes has not been studied yet.

Novelty in the Work: All the existing studies designed the socket programming-enabled healthcare systems [1,2,3,7,9,10,12] presented in fog cloud networks. The goal was to run healthcare applications on the client and offload their workloads to the fog and cloud servers for executions. In order to protect and save data, many centralized security-enabled security algorithms suggested and handled the security mechanisms in the socket client–server system for healthcare applications. However, there are many issues with the socket based-security methods, including centralized security methods that can only be implemented on a single node where sockets have mobile, fog, and cloud networks. These are autonomous nodes, and it is very hard to manage security with different rules in the system across all different nodes; it incurs the high processing cost, network cost, and storage requirements of the system. Bockchain technology, however, is an emerging technology that can support decentralized security, but it still has not been implemented in socket programming-based healthcare systems for the healthcare applications.

Motivation: Socket RPC is a widely used architecture to develop client–server architectures based on healthcare applications in practice. The existing socket-based RPC for healthcare applications is straightforward and supports the client–server architecture. However, a single centralized controller controls all the components in the current socket RPC. Therefore, the existing systems, with limitations such as a single point of failure, low security, and high processing and storage costs, are the most significant issues in the existing socket RPC architectures for healthcare applications. This study devised a decentralized, cost-efficient, secure, and potent blockchain-enabled socket RPC for healthcare applications, which is more optimal for healthcare applications than the existing architectures developed by other studies.

This study devises a potent blockchain-enabled socket RPC Internet of Healthcare Things (IoHT) framework for medical enterprises (e.g., healthcare applications). The goal is to minimize service costs, blockchain security costs, and data storage costs in distributed mobile cloud networks. The study considered the following healthcare enterprises at work: heartbeat, blood pressure, and remote counseling applications. These are coarse-grained workloads that can be executed and offloaded to the fog node for execution. The study considers the heterogeneous fog nodes implemented at the edges of hospital networks. The study was designed using socket programming-based blockchain technology, and makes the following contributions to the literature.

- Potent Socket Blockchain: This study also devises a potent (e.g., robust or lightweight) blockchain-enabled RPC based on socket programming. The study designs the RPC based on blockchain rules where a coarse-grained workload encrypts and decrypts with public and private keys based on asymmetric rules. The proof of work for the blockchain method is integrated inside socket programming to ensure the security of replica data and node-to-node validation in the system;
- Client-Socket Blockchain: This study designed the blockchain mechanism in the client socket. Each data processes the blockchain rules and offloads data to the server socket via JavaScript Object Notation (JSON). The client-socket blockchain can monitor and encrypt/decrypt data based on the available resources in the system;
- Server-Socket Blockchain: The server socket is a generous block that creates the mines (e.g., blockchain blocks) and communicates their data based on a JSON socket. The server-socket blockchain is a manager that handles server and client data security and replication in the system;
- Proof of Work: The study devises a socket-enabled proof [14] of work (PoW) and an advanced standard encryption (AES) [15] method, ensuring data replication, security, and validation inside the blockchain-enabled socket RPC for healthcare applications;
- Scheduling: Each application has a deadline and processing method, and each fog node has a resource cost and storage cost constraint. Therefore, the study devises a scheduler that maintains the quality of service of the applications and fog nodes inside the system with the suggested scheduler method.

The rest of the paper is organized in the following way. Section 2 discusses the related work. Section 3 explains the proposed blockchain-enabled socket RPC and the problem formulation. Section 4 defines the algorithmic framework for the problem. Section 5 shows the simulation results and discussion. Section 6 is the conclusion of the study. Section 7 illustrates the future work of the study for the next work.

## 2. Related Work

The client–server architecture is one of the most widely exploited mechanisms for distributed applications. A thin client makes the request, and a thick client processes the request according to a given set of requirements without degrading its performance. The socket is an open application programming interface that offers many classes to design decentralized distributed applications for medical applications. The remote process call (RPC) architecture is the best example of socket programming for medical applications. Many studies suggest medical care enterprise architectures and systems based on typical object request broker architectures (CORBAs), remote method invocations (RMIs), and service-oriented architectures (SOAs).

For instance, Refs. [1,2,3] investigated CORBA architecture-based medical systems based on an advanced encryption standard method (AES) to offload data from local devices to the remote servers for execution. Transport-level security was managed in these studies. However, other layers, such as the data link and different security layers, did not support the CORBA model. Refs. [4,5,6,7] suggested a procedure call based on the service-oriented architecture in distributed cloudlet networks for medical applications: the mobility- and location-aware healthcare services developed inside the architecture for mobile patients at work. Message digest security is considered during the data migration and offloading in the system.

Ref. [1] devised a CORBA-enabled client–server architecture based on the message digest (MD5)-enabled method in a distributed mobile cloud network. The goal was to offload the workload from applications that are based on MD5 and executed on the cloud node and send their results back to the mobile devices. This work considered the heartbeat healthcare workload for the simulation in the system. The blood pressure (Blood-P)-enabled workload is offloaded in the client–server architecture in the mobile cloud network with a secure hashing algorithm (SHA-256)-based security that was suggested in [2]. The goal was to minimize the security risk in the mobile cloud network. This study designed a client–server architecture based on remote RPC in the mobile cloud system. Android X86 and Amazon mobile cloud application programming interface (API) services are widely used for simulation. Advanced encryption standard (AES)-enabled healthcare systems based on RPC in the mobile cloud were suggested in [3]. The goal was to minimize the end-to-end security in the system. The AES and Rivest, Shamir, and Adleman (RSA) algorithm based on an RMI client–server architecture was suggested by [4,5,6]. These studies all designed their RPC, RMI, and CORBA-based clients and servers in the Java and C/C++ languages. Socket programming-based client–server and peer-to-peer healthcare applications based on remote networks and Bluetooth were designed in [7,8,9,10,11]. The goal was to offload the client data to the fog cloud for execution based on centralized CRC32 in the socket network. Refs. [12,13,16,17] suggested blockchain-based offloading in the fog cloud network, as blockchain is a technology that records and validates each connected node’s transactions in the distributed network. Different blockchain frameworks, such as Ethereum, Fabric, Corda, and others, have public and private types. Refs. [18,19,20,21,22] suggested a blockchain-enabled healthcare system in which applications are offloaded to their secure workload network for execution. Each patient data transaction is replicated at different nodes and has an immutable process to avoid any attack in the system. However, existing RSA, AES, CRC32, SHA-256, and MD5 security methods are widely used in client–server servers. Furthermore, blockchain technologies protect data in different network nodes for healthcare applications. However, the existing CORBA, PRC, RMI, and SOA client–server architectures lack decentralized security models in which heterogeneous nodes can connect easily and transfer data in the form of JavaScript Object Notations (JSON) across the mobile edge cloud network.

To the best of our knowledge, the decentralized security-enabled client-server system for healthcare applications in mobile fog networks has not been studied yet. The work presents a potent blockchain-enabled socket RPC Internet of Healthcare Things framework for medical enterprises. The goal is to reduce healthcare applications’ service, blockchain, and storage costs in a client–server framework. The study devises a socket programming-based blockchain-enabled RPC framework for healthcare applications in which different costs improve during execution in the framework. The existing blockchain frameworks, namely, Ethereum- and Fabric-based RPCs, focus only on financial applications and cannot be applied directly to healthcare applications. Simulation results show that the proposed blockchain-enabled socket RPC minimized the service cost by 40%, the blockchain cost by 49%, and the storage cost by 23% for healthcare applications. Table 1 shows the mathematical symbols of the problem and their description.

## 3. Proposed System

**Figure 1:**
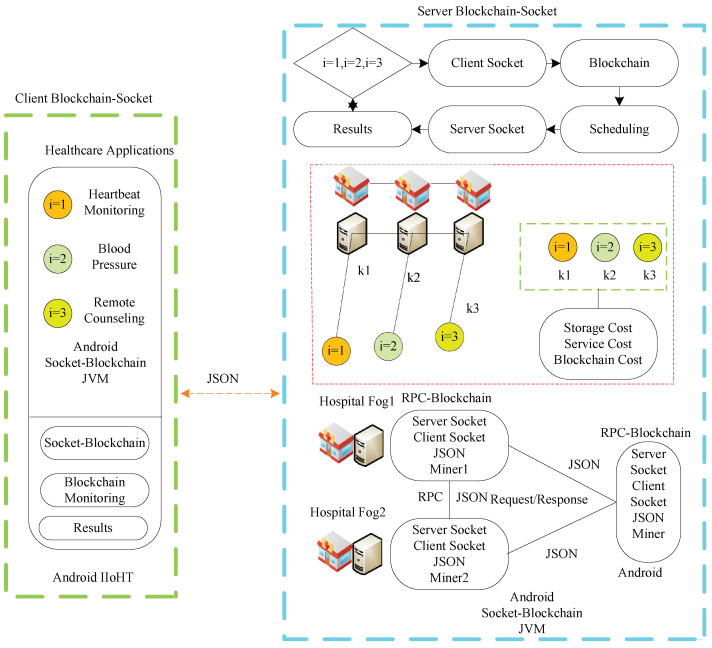
Potent blockchain-enabled socket RPC-aware IoHT framework for medical enterprises.

The study devises a potent blockchain-enabled client–server system based on distributed fog networks to run the different IoHT components in the framework. The proposed system is more flexible to support other Internet of Healthcare Things applications in the network. The study devises an IoHT framework that consists of different layers. There are two main layers, the client-server blockchain and the server-socket blockchain, for medical enterprise applications, as shown in Figure 1.

Three healthcare applications are implemented on the client-socket blockchain: heartbeat monitoring, blood pressure, and remote counseling. These are coarse-grained applications represented by the notations {i=1,i=2,i=3}. The client socket offloads entire workloads to the server socket for processing. The client socket is implemented inside mobile devices where workloads are transactions, called the genesis blockchain. The mobile device has an Android X86 JVM runtime environment, where blocks and socket programs can be developed in the Java language, as shown in Figure 1. Initially, all the workloads are processed locally by the blockchain. Their transactions offload their workload to the server socket for processing.

The server socket layer is the processing layer where all requested workloads are executed under requirements, as shown in Figure 1. The three different workloads represented by {i=1,i=2,i=3} are allocated to the three different computing processes and are represented by {k=1,k=2,k=3}. The computing nodes are fog-implemented at different hospitals based on remote procedure calls. The workload offload is based on the JSON protocol between a client and the server in the system.

### Problem Formulation

The study considered three heterogeneous coarse-grained healthcare workloads: blood pressure, heart monitoring, and remote counseling. Each workload has a particular deadline, e.g., $i_{d}$. The study considered three different heterogeneous fog nodes, e.g., {k=1,k=2,k=3}. Each fog node has a particular speed, e.g., $speed_{k}$, and resource limit, e.g., $k_{r}esource$. The mobile devices are annotated by m=1,m=2,m=3; each mobile m1 has limited speed and resources, $m_{s}peed$ and $m_{r}esource$, respectively. In our case scenario, the study considers the three different hospitals, e.g., h1,h2,h3, where all fog nodes are implemented in all individual hospitals in the proposed work. The service cost of the workload is determined in the following way.

(1) $$Service-Cost=\sum_{m=1}^{M}\sum_{i=1}^{i=2,3}\sum_{k=1}^{K}\frac{i}{m_{s}peed∼speed_{k}}\leq i_{d}\times x_{h,i,k}=1.$$

In Equation (1), $x_{i,k,s}$ is the binary variable that denotes the allocation of the workload on the particular server and exploits particular storage. Equation (1) determines the service execution cost of all workloads in the system. This study implements the potent blockchain technology inside the RPC socket-based framework. Therefore, the blockchain cost is determined in the following way:

(2) $$Blockchain-Cost=\sum_{m=1}^{M}\sum_{h=1}^{h=1,2}\sum_{b=1}^{b=2,3}\sum_{i=1}^{i=2,3}\sum_{k=1}^{k=2,3}b,i,m,k.$$

Equation (2) determines the blockchain cost, which is equal to b←<hashing,previous-hash,PoW,timestamp>, where *b* is the particular blockchain block that has different attributes; the hashing hashing is conducted based on AES-256, and previous hashes match the hash during the workload transaction as well as the timestamp of the transaction timestamp. The study considered different cloud storage methods, such as s1,s2,s3, for the workload results’ savings, and their cost was determined in the following way:

(3) $$Storage-Cost=\sum_{s=1}^{S}\sum_{i=1}^{i=2,3}\sum_{k=1}^{k=2,3}s,i,k.$$

Equation (3) determines the storage cost, such as s1,s2,s3, attained using Dropbox, Google Drive, and Microsoft One Drive. Each data storage method has a different cost in the framework; all the storage methods are heterogeneous. The problem is mathematically formulated in the following way:

(4) minService-Cost,Blockchain-Cost,Storage-Cost,∀i=1,…I.

Equation (4) determines the objective function of the study, subject to:

(5) $$\sum_{i=1}^{I}\frac{i}{\zeta_{m}}+\frac{i}{\zeta_{k}}\leq d_{i},\;\forall i=1,\ldots ,I.$$

Equation (5) ensures that all workloads must be executed under their deadlines at both the client socket and the server socket during processing in the system.

(6) $$\sum_{i=1}^{I}\frac{i}{\zeta_{m}}+\frac{i}{\zeta_{k}}\leq \epsilon_{m,k},\;\forall i=1,\ldots ,I.$$

Equation (6) ensures that all the processing client and server nodes must have sufficient resources to run the workloads at both the client and server sockets during processing in the system.

## 4. Socket-Blockchain RPC Algorithm Framework

The study devises a socket-blockchain RPC algorithm framework in which the socket process is based on the blockchain employed in the work.

The proposed algorithm (Algorithm 1) is the main framework, and the algorithm’s framework has the following steps:

- Initially, all the workloads and resources are input in Algorithm 1;
- All the workloads are sorted according to their deadlines, as defined in steps 2 to 3;
- The available fog nodes in the particular hospital *h* are serched, based on their available resources and speed in the framework defined in step 3;
- From steps 6 to 9, Algorithm 1 starts the process from the local mobile device to the fog node based on the socket RPC in the system. The service cost process is applied based on Equation (1);
- From steps 12 to 17, Algorithm 1 applies blockchain technology to design the work in socket programming. In the blockchain process, the algorithm generates the hashing based on SHA-256 and uses the local transaction from the mobile device to the fog node as the hash. The previous transaction hash must be matched in the fog node hash before being processed for storage in the fog node. The study verifies the proof of work (PoW) to validate the data in the framework;
- The storage is conducted in steps 18 to 20 of the executed workload in the system. In the framework, the study chooses the lowest-cost storage for the workload execution results.

**Algorithm 1:** Socket-Blockchain RPC Algorithm Framework
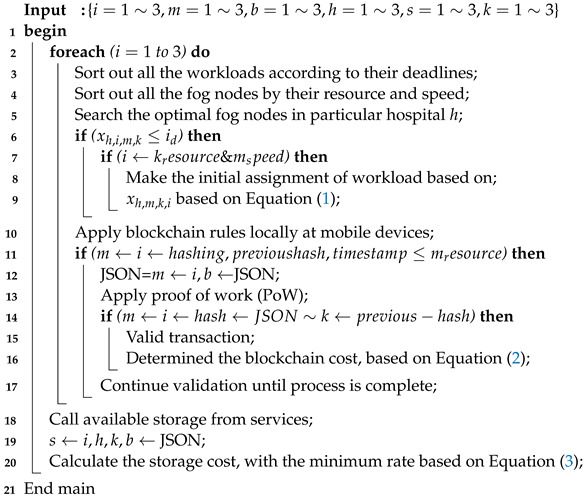


### 4.1. Blockchain-Enabled Android Socket Layer

The study devised a client-socket blockchain method, which takes the healthcare workload as input and applies blockchain rules inside the head. The study involves the hashes of the data based on the AES (advanced standard encryption) algorithm and generates random alphanumeric public and private keys. The study devises the client-socket blockchain-enabled Algorithm 2, which takes the following steps to run the data.  
**Algorithm 2:** Client-Socket Blockchain Scheme
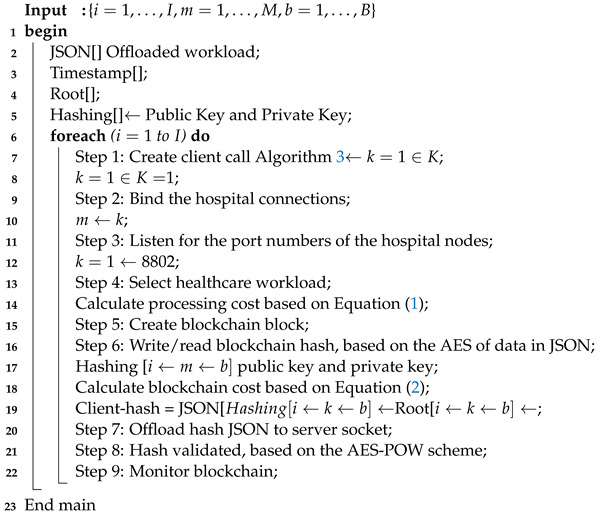

**Algorithm 3:** Server-Socket Blockchain Scheduler
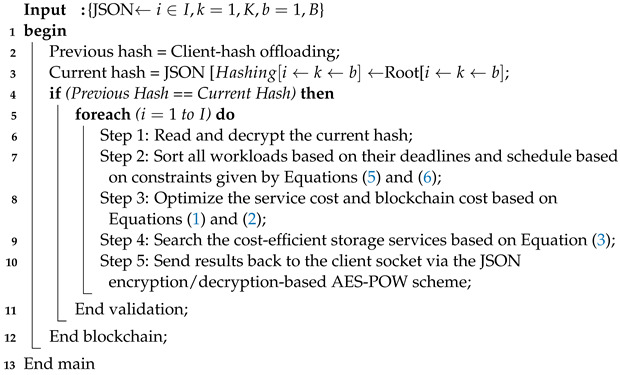


**Figure 2:**
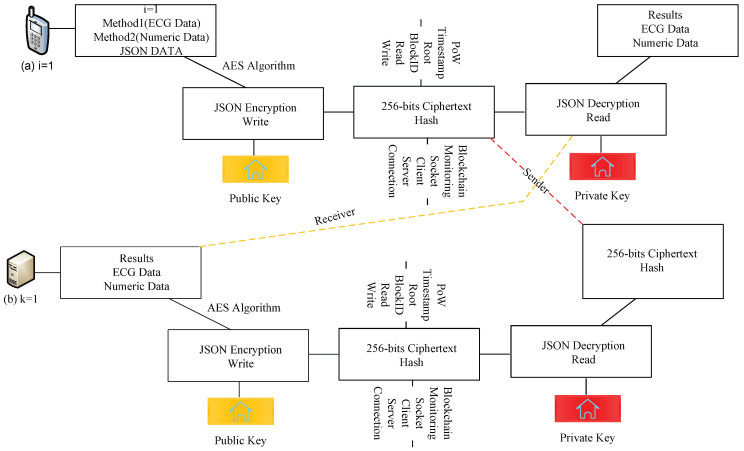
(**a**) Client-socket blockchain; (**b**) server-socket blockchain.

The blockchain manager and blockchain monitoring system are both designed functions that exist on both the client and the server sockets; they can add any block and monitor it. The study encrypts and decrypts data inside the blockchain block based on the AES public and private keys in both client and server sockets inside the model. The blockchain manager creates the particular block with each application’s unique identification (ID). Each blockchain, e.g., *b*, has a blockID, a timestamp, an AES-256-bit hashing, a root, and the data transactions of the application functions inside the block. Figure 2 shows the process of the client-socket blockchain and the server-socket blockchain during offloading and execution in the system, whereas Figure 2a,b shows the client and server sockets that accept the coarse-grained application. Each workload is encrypted in the blockchain block based on advanced standard encryption (AES) ciphertext that is based on a 256-bit public key. The proposed security method is lightweight and has low time and space complexity during processing inside blockchain blocks for the hashing of workloads in the system. The cipher is offloaded from one node to another for the data transaction. It has a validation based on the system’s proposed proof of work method to avoid tampering or attacks at different nodes. In this work, the validation study, where valid data has been transmitted between nodes during processing for the execution, is the highest priority. For instance, the workload is executed on the fog nodes and the executed results are stored in the cloud for cost-saving purposes. Therefore, the data transmission among nodes must be validated and transferred accurately without any tampering.

**Figure 3:**
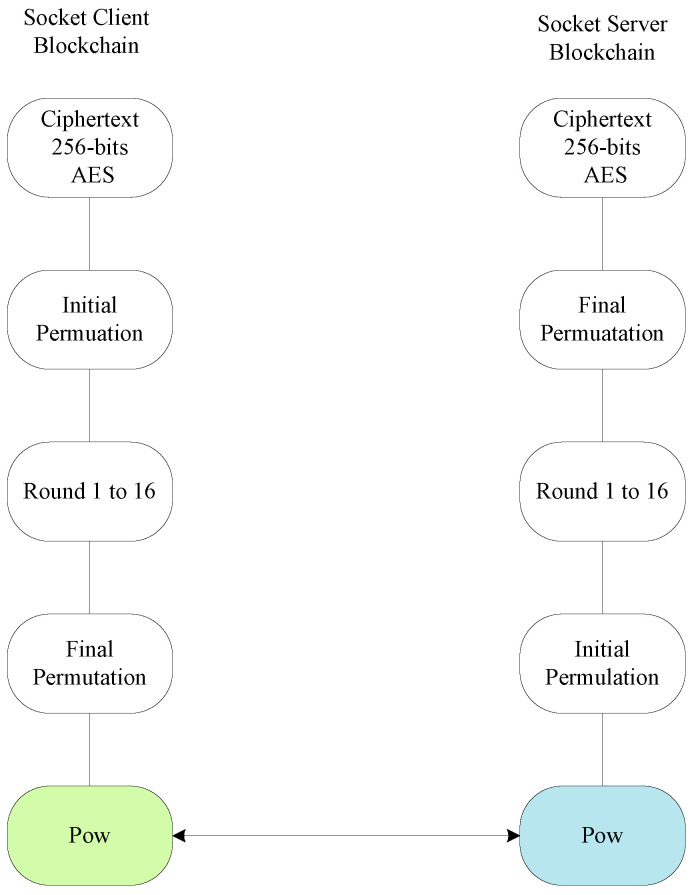
DES-enabled PoW scheme in blockchain-enabled nodes for healthcare workloads.

The AES algorithm is mixed with the proof of work (PoW) such that the initial permutation makes 48 rounds to make the secure key, keep the security, and authenticate the record in the particular blockchain block at the client side, as shown in Figure 3. The final permutation holds the generated (e.g., write) 256-bit ciphertext and offloads it to the server-socket blockchain. The PoW on both sides validated the ciphertext data at different timestamps between two client and server blocks for offloading and processing data in the system. The 48 rounds of key permutation generated the strong key and kept the encryption data with a very authentic and immutable inside block.

Algorithm 2 creates a blockchain connection to an available server block based on Algorithm 3. Once the connection is established, the fog node-enabled hospital connection is bound and establishes the connection on the particular port number as it listens to the command. The coarse-grained healthcare workload process is based on its functions, such as heartbeat function, monitoring, and reporting, and each function encrypts and decrypts (writes and reads) based on the AES inside the block. The ciphertext-encrypted data is offloaded via JavaScript Object Notation (JSON) to the server socket. The hash validation between the client and server sockets on JSON is to be conducted based on the proof of work (PoW).

### 4.2. Blockchain-Enabled Fog Socket Layer

Algorithm 3 is the server-socket blockchain scheme that runs the workloads based on the offloaded JSON hashes and matches the current and previous hashes at the designated timestamp on the particular root. After the validation of the hash between the client and server sockets, the method decrypts the AES-based private key, as defined in step 1, and sorts the workloads based on their deadlines, scheduling them as defined in step 2. The server socket searches for the optimal and most cost-efficient data storage service to save the workload results, and sends them back to the client socket, as defined in steps 3–5.

## 5. Performance Evaluation

The study designed a potent blockchain-enabled client- and server-socket remote procedure call simulator and application based on advance Java application programming interface (API). The simulation parameters that are implemented in the potent blockchain-enabled socket RPC Internet of Healthcare Things framework for medical enterprises are defined in Table 2.

The notations of the simulator parameters and their usage are already discussed in the problem formulation and in the algorithm designed in the study.

Table 3 shows the node cost in the simulation, and has a different cost assigned to each type of node.

### 5.1. Implementation

Based on JAVA socket programming, the study designs a potent blockchain-enabled client–server remote procedure call (RPC). The study designs the simulation based on applications and implements all fog services based on socket programming. In this work, the simulation was conducted based on the Android X-86 emulator, and Cisco fog nodes were implemented based on socket programming.

### 5.2. Numerical Data Analysis

Table 4 shows the numeric values of all existing architectures with their considered constraints in the simulation work for the healthcare applications. These architectures obtained their results based on execution times and resource service costs. The deadline is the constraint determined based on processing costs and failure recovery costs in the simulation environment. Table 4 shows that the proposed potent socket client–server blockchain-enabled RPC is more optimal and offers decentralized security, with the hashing validation and by adding blocks with immutable attacks in the system, as compared to existing centralized security models. The client and server sockets run on the same platform, such as the proposed system’s Java virtual machine (JVM). The JVM is a flexible and open-source environment and supports all of the file classes that client and server sockets have in the system. All the parameters of Table 4 are defined in the following.

- The study integrated an SOA architecture for the performance evaluation of the system, where all the parameters are considered in the system. SOA is a collection of class libraries where classes support client and server architectures;
- Values of 1300 (ms), 700 (ms), and 700 (ms), blood pressure, and an execution cost of 1200 (ms) are implemented with the CORBA architecture for the healthcare applications in the system;
- Values of 1500 (ms), 700 (ms), and 800 (ms), ECG heartbeat, and an execution time of 1200 (ms) are implemented with the RMI architecture for the healthcare applications;
- RPC is the architecture that offers a client–server architecture based on socket programming in the system. The applications are executed on different nodes with all architectures, where cost and execution time are parallel-optimized in the system.

### 5.3. Results Discussion of Healthcare Applications Based on Different Schemes

In this section, the study determined the cost performances (e.g., those that are calculated in USD) of the methods for the coarse-grained healthcare workloads in the system. In subpart, the study discusses the different schemes that executes the healthcare workloads on the distributed computing nodes. The study discusses the processing cost, blockchain cost, and storage cost performance of methods for the coarse-grained healthcare workload in the system. The coarse-grained healthcare workloads in this study were only assigned to more than one node for the nodes during execution to minimize their costs in the system. The service cost is a logical cost of the workload execution in a socket-based RPC framework. Socket programming has different steps, such as client-socket and server-socket steps. The study implemented the remote procedure call services with the designed blockchain technology in the implementation part. The study implemented the existing baseline approaches, such as SHDS [12], RPC-Medical [9], and the proposed blockchain-socket RPC, in the system.

**Figure 4:**
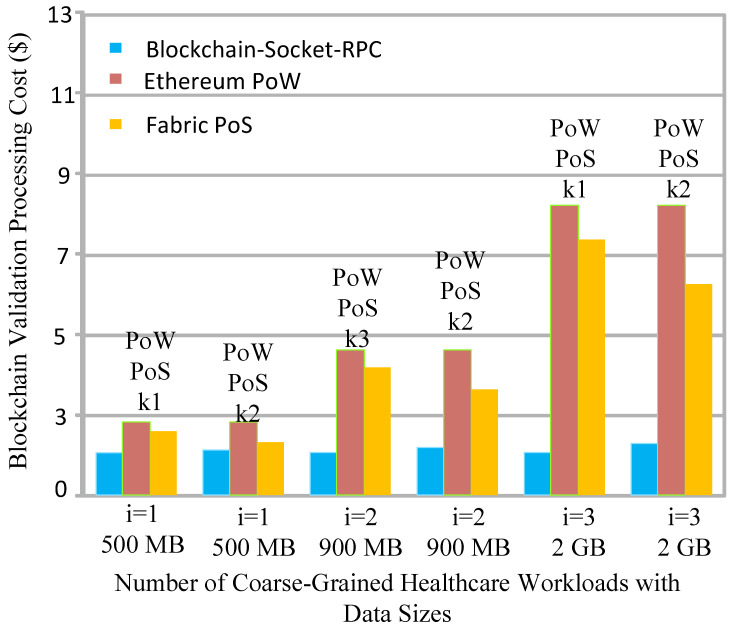
Potent Blockchain processing cost during the processing of workloads on heterogeneous computing nodes.

Socket programming has a peer-to-peer network, again, in terms of a client–server architecture; in RPC, we considered the different nodes, and each workload was offloaded to the one fog node. The blockchain process is completed on the same machine where the initial execution, as well as the available free space, was performed with the minimum hashing and proof of work validation in the blocks. All the workloads were hashed based on SHA-256, and made transactions between a mobile device-client socket and a server-socket fog node for the processing. The consensus algorithm proof of work validated each transaction, from execution to storage, in this work. The existing Ethereum [11] and Fabric [12] blockchains for distributed healthcare applications have a lot of validity and security. However, Ethereum and Fabric were initially designed for financial applications and need a lot of resources for execution. Figure 4 shows that the proposed blockchain socket RPC is more efficient in terms of cost, as compared to the existing one.

**Figure 5:**
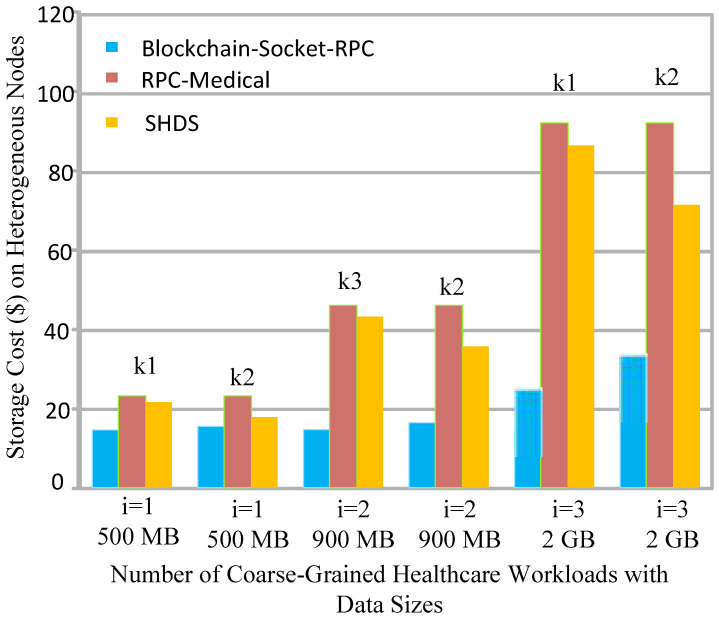
Storage cost of the number of healthcare workloads.

Each hospital offers different storage options in the framework, as shown in Table 3. Figure 5 shows that the optimal storage from the existing third-party provider suffers from resource leakage due to their limited resource availability in their fog nodes. There is a valid reason for this, and all existing socket-based healthcare systems have offloaded all workloads to fog computing or cloud computing for processing. The fog nodes have scalability issues, and cloud computing has high latency issues. Therefore, existing methods and systems incur issues of scalability, resource leakage, high latency, and cost, as shown in Figure 5. The results discussion shows that processing on the fog nodes and their storage on the cloud minimized the cost and processing as well as the costs of the healthcare applications in the healthcare system. Therefore, the proposed idea with the socket-blockchain architecture obtained the optimal results for the healthcare applications and utilized resources in the optimal ways, as shown in Figure 5.

**Figure 6:**
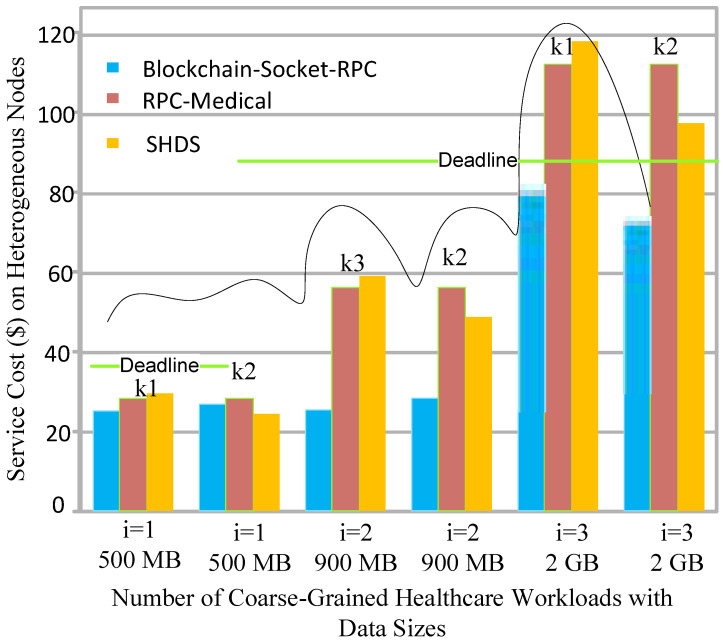
Service cost of the number of healthcare workloads.

Figure 6 shows that the proposed socket-blockchain RPC has less service cost as compared to existing socket-based methods and RPC-based methods for healthcare applications. The main goal is to divide the workload between different nodes as existing workloads were executed on the same node, which incurs an extra cost stemming from the execution of huge workloads on the same node. Then, the workload is offloaded to the particular hospital, which has different fog nodes based on their costs. All the workloads were executed according to their deadlines. In Figure 6, the green lines show the fixed-threshold deadline before the execution of the workload in the system, whereas workload i=1 and workload i=2 somehow meet the deadlines with all methods. However, workload i=3 and workload i=4 missed the deadline. The main reason for this is that all existing methods only considered the client node and server, where the fog node incurred resource scarcity issues and the cloud incurred the higher latency issue. Therefore, in both cases, the deadline was missed in the system with the existing studies for the healthcare applications. The proposed work balances the load between the fog and cloud, and minimizes the overall costs, meeting the deadline of the applications during their processing in the system.

## 6. Conclusions

The study devised a socket programming-based blockchain-enabled remote process call (RPC) framework for healthcare applications in which different costs improve during execution in the framework. The existing blockchain frameworks, namely, Ethereum- and Fabric-based RPCs, focus only on financial applications and cannot be applied directly to healthcare applications. Simulation results showed that the proposed blockchain-enabled socket RPC minimized the service cost by 40%, the blockchain cost by 49%, and the storage cost by 23% for healthcare applications. The results discussion part showed how all workloads are executed on different computing nodes and meet the quality-of-service requirements with the other methods. Initially, the study compared the performance of the existing blockchain technology schemes, such as proof of work and proof of stake, with the proposed socket-blockchain RPC algorithm. The results discussed showed that the proposed blockchain framework-based validation scheme obtained optimal results compared to existing blockchain schemes. The main reasons for this were the optimal usage of resources and the low storage costs of the workloads, with the minimum costs in the system. In the proposed work, the study validated the data on a node-by-node basis with the proposed lightweight hashing method, which is lighter than proof-of-work and proof-of-stake methods. Moreover, in our proposal, we processed and executed the workload data on the fog node and stored the data in the cloud with the cheapest storage cost in the system. The results of the discussion showed that the proposed work is more optimal and obtains better results than existing methods for the healthcare workloads in the system.

## 7. Future Work

In future work, the study will optimize the energy efficiency of the mobile device, fog nodes, and blockchain processes in the framework for healthcare applications. The study will consider the mobility healthcare services with the different security constraints for the healthcare applications. The study will design the mobility-enabled proof-of-work methods for the distributed blockchain networks for the healthcare applications.

## Figures and Tables

**Table 1:** Formulation Mathematical Notations.

Notations	Description
*I*	Set of coarse-grained healthcare workloads
*i*	Healthcare enterprise workload *i*
$d_{i}$	Deadline of workload *i*
*M*	Number of client nodes
*m*	Particular node, such as mobile node
$\epsilon_{m}$	Resources of particular node
$\zeta_{m}$	Speed of node *m*
*K*	Number of socket servers
*k*	Particular node, such as fog node *k*
$\epsilon_{k}$	Resources of particular node
$\zeta_{m}$	Speed of node *k*
*B*	Shows the size of blocks
*b*	Particular block deployed at any node
*S*	Amount of storage available
*s*	Particular storage

**Table 2:** Potent blockchain-enabled RPC framework.

Configuration Parameters	Parameter Values
IoHT devices	Android X-86 Phones
IoHT devices	Android-Emulator Phones
Platform	Socket JAVA Virtual Machine (JVM)
Cisco Fog platform [23]	Fog nodes’ implementation
Socket-Programming API	JAVA
i=1	200 MB heartbeat workload
i=2	900 MB blood pressure
i=3	2 GB MB remote counseling
m=1	Android—latest version
k=1	Core I5 30 GB RAM
k=2	Core I7 100 GB RAM
k=3	Core I9 500 GB RAM

**Table 3:** Processing Cost of Computing Nodes.

Node	Cost
s=1 (Dropbox)	USD 2 per hour
s=2 (Google Drive)	USD 3 per hour
s=3 (One Drive)	USD 0.5 per hour
k=1	USD 1 per hour Core (I5 30 GB RAM)
k=2	USD 2 per hour Core (I7 100 GB RAM)
k=3	USD 3 per hour Core (I9 500 GB RAM)

**Table 4:** Existing client-server architectures and Their Constraints.

Architecture	Service Cost	Security Cost	Storage Cost	Workload	Deadline
SOA	1000 (ms)	600 (ms)	500 (ms)	Heartbeat	1200 (ms)
CORBA	1300 (ms)	700 (ms)	700 (ms)	Blood Pressure	1200 (ms)
RMI	1500 (ms)	700 (ms)	800 (ms)	ECG Heartbeat	1200 (ms)
RPC	1600 (ms)	900 (ms)	800 (ms)	Heartbeat	1200 (ms)
Proposed-Work	500 (ms)	200 (ms)	300 (ms)	Heartbeat	1200 (ms)
**Architecture**	**Service Cost (USD)**	**Security Cost (USD)**	**Storage Cost (USD)**	**Workload**	**Deadline (USD)**
SOA	10	60	50	Heartbeat	12
CORBA	13	70	60	Heartbeat	15
RMI	18	88	45	Heartbeat	11
RPC	40	20	40	Heartbeat	19
Proposed Work	3	6	5	Heartbeat	5

## Data Availability

Data is a private and can not be shared publically in the first phase.
